# Development and evaluation of a new assistive device for low back load reduction in caregivers: an experimental study

**DOI:** 10.1038/s41598-022-21800-5

**Published:** 2022-11-09

**Authors:** Yuka Omura, Masayuki Hirata, Toshiki Yoshimine, Eiji Nakatani, Tomoko Inoue

**Affiliations:** 1 Division of Health Sciences, Osaka University Graduate School of Medicine, Osaka, Japan; 2 Global Center for Medical Engineering and Informatics, Osaka University, Osaka, Japan; 3 Department of Neurological Diagnosis and Restoration, Osaka University Graduate School of Medicine, Osaka, Japan; 4 Endowed Research Department of Clinical Neuroengineering, Global Center for Medical Engineering and Informatics, Osaka University, Osaka, Japan; 5 Iseikai Medical Corporation, Osaka, Japan; 6 Graduate School of Public Health (Medical Statistics), Shizuoka Graduate University of Public Health, Shizuoka, Japan; 7 Division of Clinical Biostatistics, Research Support Center, Shizuoka General Hospital, Shizuoka, Japan

**Keywords:** Health services, Occupational health

## Abstract

Low back pain among healthcare professionals is associated with the manual handling of patients. Some bed features for turning and repositioning have been developed; however, the load during patient care remains heavy. We developed a device to reduce low back load in caregivers during patient bedside care and evaluated it objectively and subjectively from a caregiver’s perspective using a randomised crossover study. Overall, 28 clinical nurses and care workers were randomly assigned to two interventional groups: administering care with (Device method) and without (Manual method) the device in an experimental room. We measured the caregiver’s trunk flexion angle using inertial measurement units and video recording during care and then defined a trunk flexion angle of > 45° as the threshold; the variables were analysed using linear mixed models. Subsequently, participants responded to a survey regarding the usability of the device. Trunk flexion time and percentage of time were 26.5 s (95% confidence interval: 14.1 s, 38.9 s) (*p* < 0.001) and 23.0% (95% confidence interval: 16.4%, 29.6%) (*p* < 0.001) lower, respectively, in the Device group than in the Manual group. Furthermore, caregivers evaluated the care they could administer with the device as being better than that associated with manual care.

## Introduction

Ageing is a major issue in many countries^1,2^. As the population ages, consequent shortages in nursing personnel become a serious problem^3,4^. Certain types of care for bedridden patients, such as repositioning the patient’s body or turning them to change diapers or sheets, impose a heavy burden on caregivers^5^. This can cause severe low back pain. Patient handling is a risk factor for the development of low back pain^6^.

Thus, more than 50% of clinical nurses and healthcare workers experience low back pain^7,8^. The prevalence of low back pain, including work-disabling low back pain, is higher in nurses than in people from other occupations^9–11^. Hence, patient-handling guidelines indicate the use of assistive devices or the cooperative efforts of two or more caregivers^12,13^, but caregiver shortages make these guidelines difficult to follow. Nurses working in hospitals and nursing homes, especially in Japan with an ageing population, often performed nursing care such as transfers and repositioning by one person^7,14^.

Recently, some devices for repositioning and turning have been developed and made available commercially^15^ to reduce caregivers’ physical stress associated with turning and repositioning patients, such as a friction-reducing turning sheet with a wedge foam^16^, a mattress with turn-assist features via inflated air bladders^15,17^, and a mattress-sized turning platform^18^. As the main purpose of these devices is to avoid pressure ulcers, they only laterally turn the care recipient. This results in a shallow tilt angle or insufficient space between the air mattress and patient’s back, which is not useful for routine activities, such as changing linens and diapers or bathing. Therefore, we developed a prototype of a breakthrough, assistive device that has been programmed to laterally turn (on the long axis of the body) and support a bedridden patient’s body during direct care.

## Development of the Programmable Lateral Position Changer

Using the bio-design approach of focusing on needs and using the most efficient methods to achieve them,^19^ we first conducted in-field observations and identified the reduction of caregiver burden in changing positions and diapers as an unmet need in the field. We then began developing assistive devices that do not require more effort than conventional care, while still using assistive devices. The ‘Programmable Lateral Position Changer’ does not require special preparation, such as placing a particular sheet under the patient’s back or setting up a mechanical lift. Furthermore, it is designed to reduce caregivers’ burden so that one caregiver can provide care, offering an alternative method of patient handling to overcome problems experienced by many caregivers using the conventional manual method, which comprises manual labour without assistive devices.

### Key features of the device

**Figure 1:**
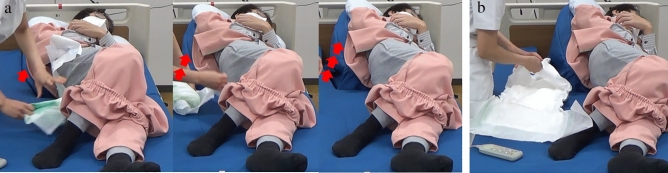
Key features of the programmable position changer. Tilting the patient’s body laterally, gradually, and automatically (**a**), and supporting the patient’s body during care (**b**).

The Programmable Lateral Position Changer has two main features. Raising part of the mattress allows the device to tilt the patient’s body laterally (Fig. 1a) and support the patient’s body (Fig. 1b) when providing care. These features could eliminate the caregiver’s physical workload during lateral tilting and reduce the amount of work required. The prototype can tilt and support the body automatically; therefore, the caregiver can provide care with both hands and does not require the help of another caregiver to stabilise the tilting body during care. This releases the caregiver from the mental burden of performing multiple tasks of physical labour and observing the patients’ health conditions during care. Furthermore this device can ensure patient’s comfort by offering a more stable support and gentle tilting method, compared to those associated with the manual method. In addition, it has customisable operation speeds and tilt angles.

### Device design

#### Description of the device

**Figure 2:**
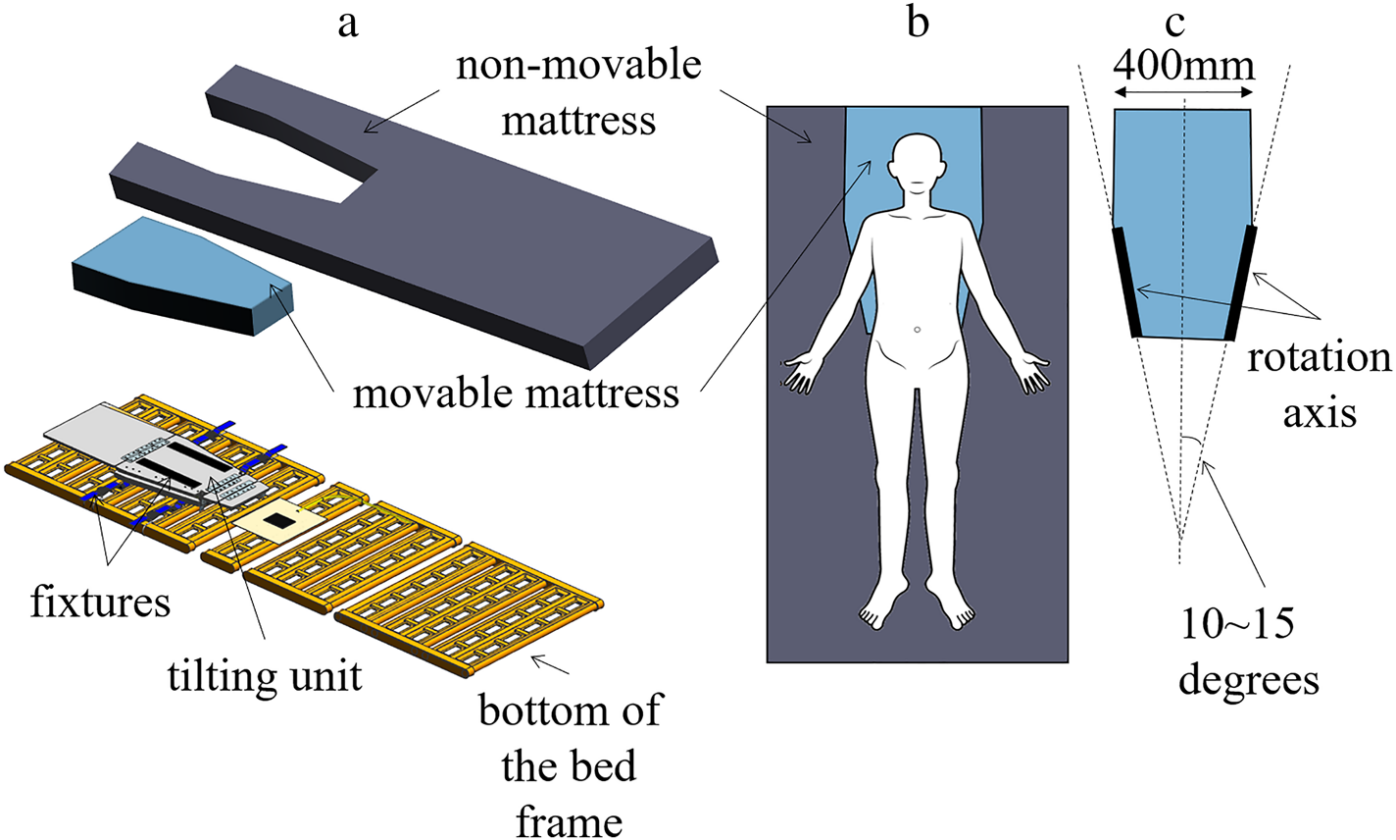
Design of the programmable lateral position changer. The device consists of a mattress and an underlying tilting unit attached to the bed frame with fixtures, and the ‘mattress’ comprises a movable and non-movable mattress (**a**, **b**). The movable mattress has a hexagonal shape, and its rotation-centre axis is tilted at an angle of 10–15° with respect to the centre line (**c**).

The device consists of a mattress and an underlying tilting unit attached to the bed frame with fixtures (Fig. 2a). The ‘mattress’ comprises a movable and non-movable mattress (Fig. 2b). The movable mattress has a hexagonal shape and is attached above the tilting unit via a fixture. It is rotated about a predetermined rotation axis by the tilting unit. The rotation-centre axis is tilted at an angle of 10 − 15° with respect to the centre line that extends in the longitudinal direction and passes through the centre of the movable plate in the lateral direction in a plain view (Fig. 2c). This angled rotation axis can facilitate natural rolling that is more comfortable for the patient. The non-movable mattress surrounds the movable mattress and is configured such that it cannot rotate. The maximum length of the movable mattress in the left–right direction is less than 2/3 of the length of the non-movable mattress. Furthermore, considering the average shoulder width of adults, the maximum length of the movable mattress in the left–right direction has been set to 400 mm.

#### The tilting mechanism

**Figure 3:**
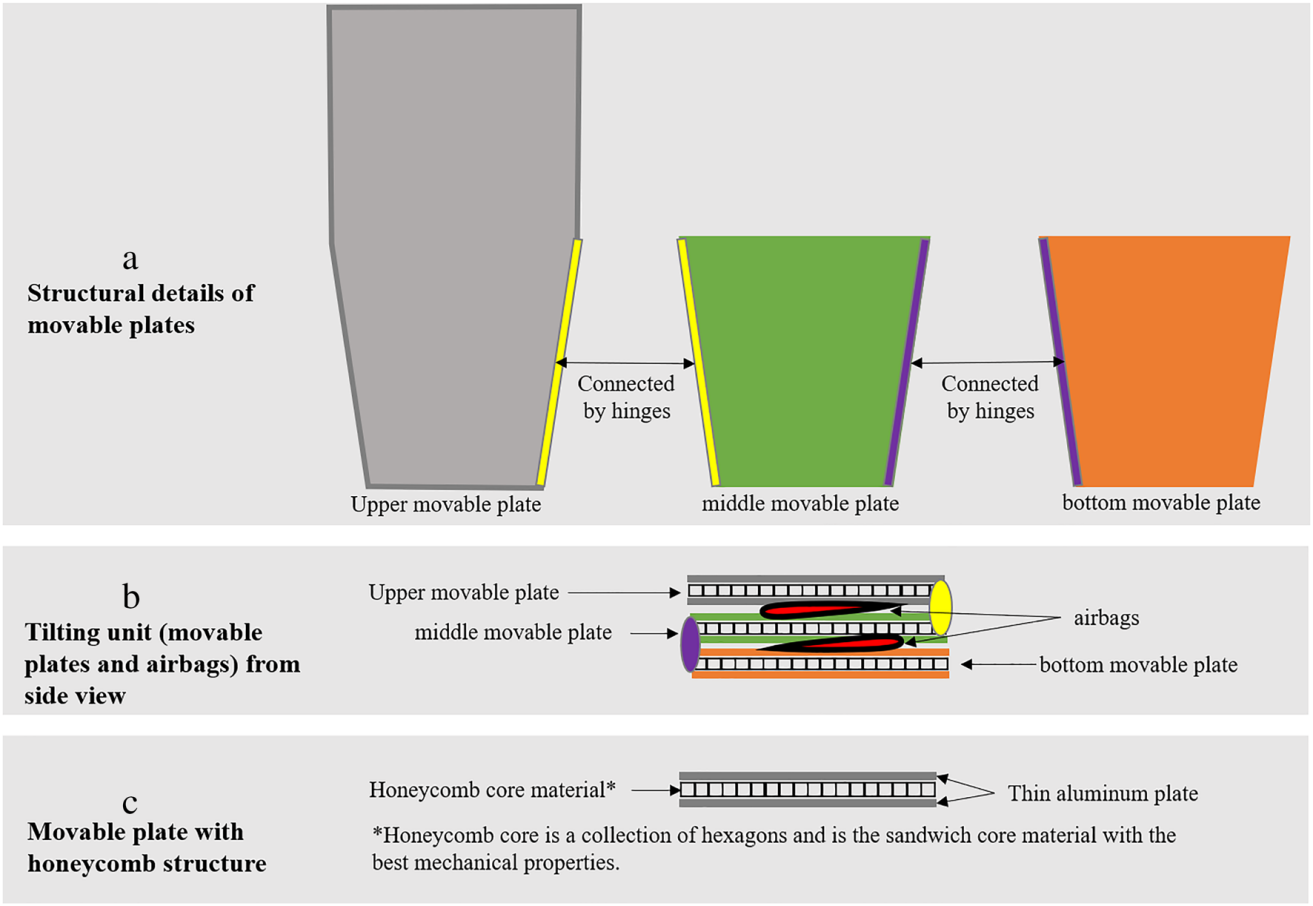
The structure of the tilting unit. The tilting device included three movable plates connected by hinges (**a**), and two inflatable/contractible airbags were placed between the hinged plates that could be folded into a Z-shape (**b**). Three movable plates, composed of a honeycomb structure sandwiched within an aluminium frame (**c**).

**Figure 4:**
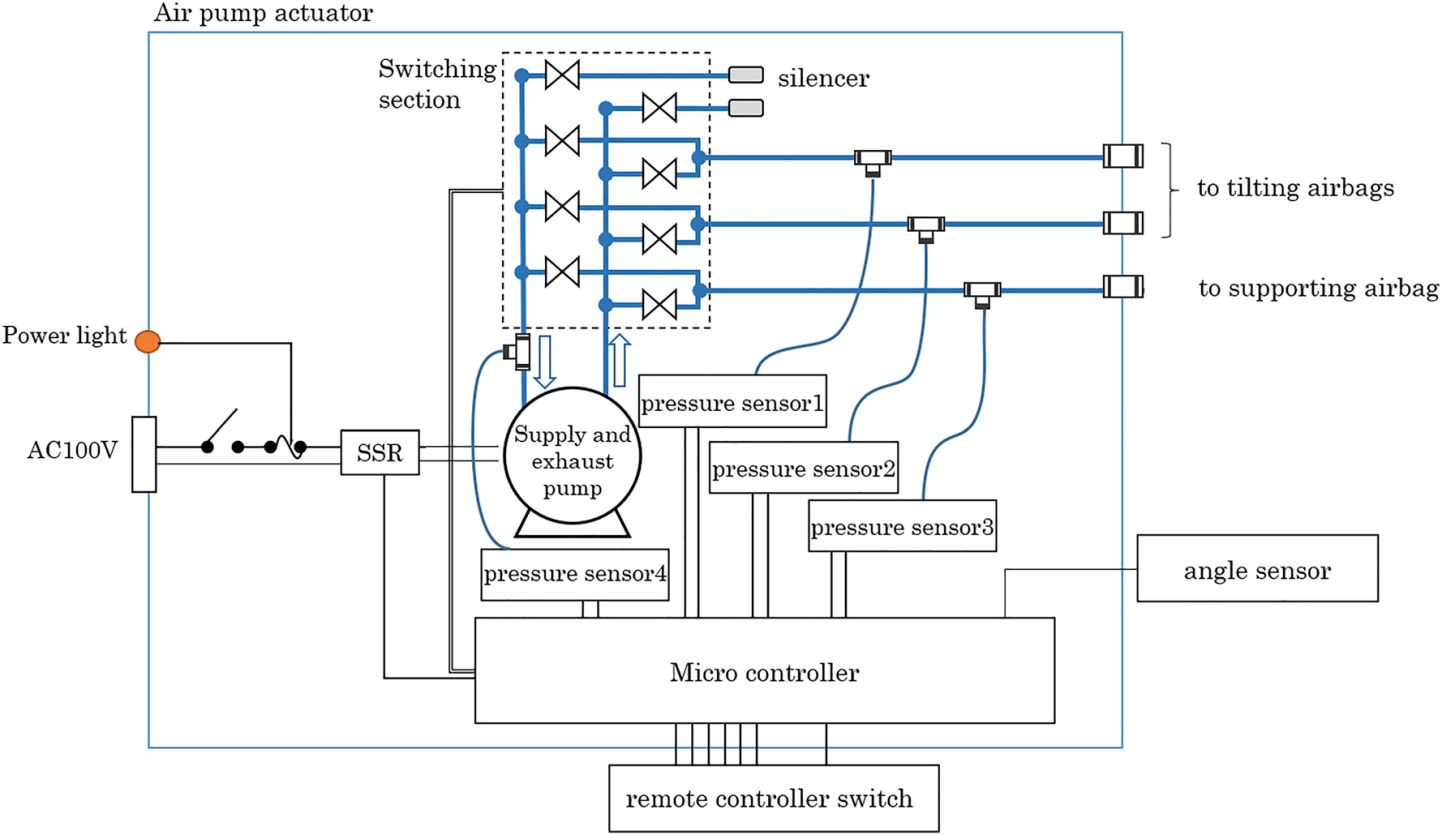
Circuit related to airbag control. The microcontroller controls the solenoid valve in the switching section and the SSR (Solid State Relay), which switches the pump on and off to achieve a specified tilting angle by detecting the tilting angle with the angle sensor.

**Figure 5:**
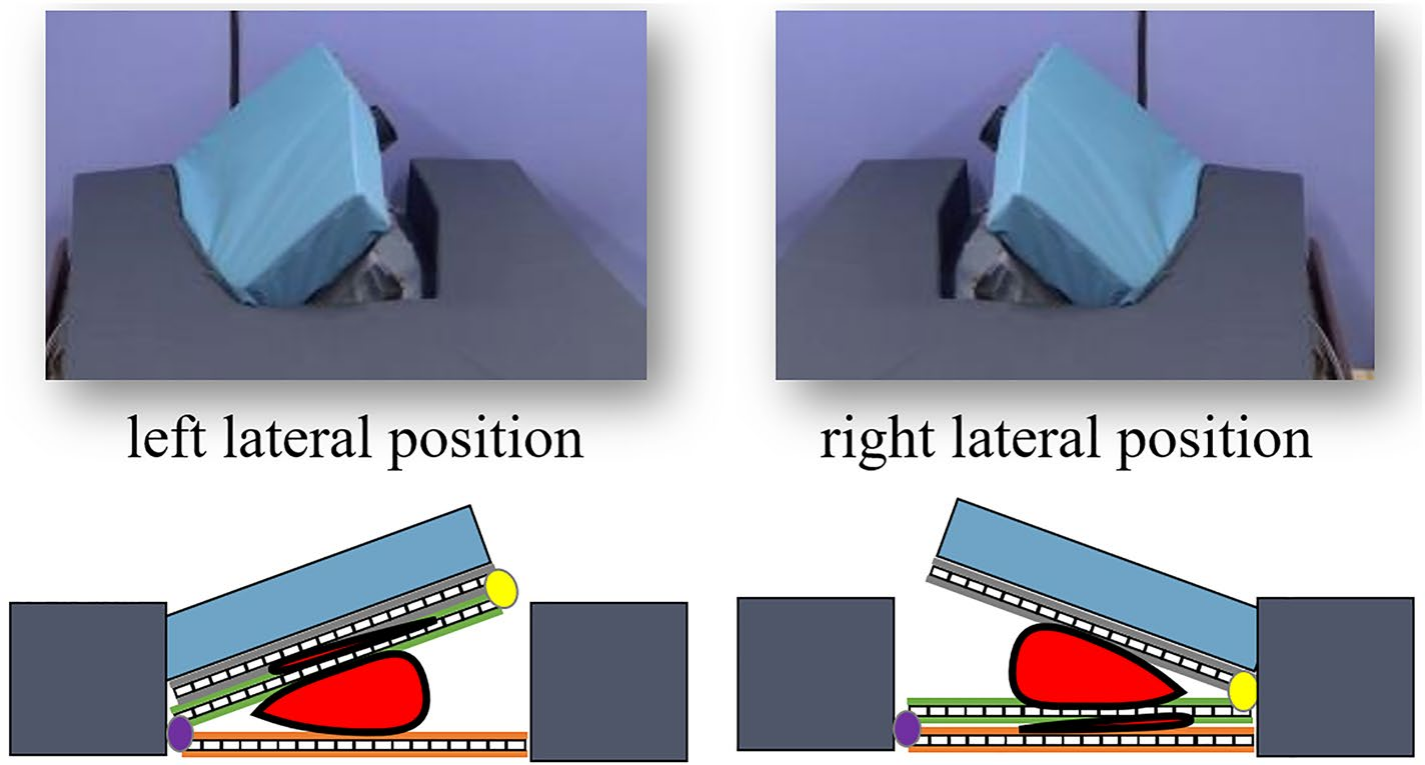
Relationship between the tilting unit and body position. The airbags assigned to each direction inflate and tilt to the left or right.

The tilting unit includes three movable plates and two inflatable/contractible airbags. Three movable plates, composed of a honeycomb structure sandwiched within an aluminium frame, have a hexagonal shape, are connected by hinges and two airbags are placed between hinged plates that can be folded into a Z-shape (Fig. 3a,b,c). The airbags can be inflated or deflated by supplying air from the air pump actuator. Figure 4 shows the airbag air control circuit. In this device, the microcontroller controls the solenoid valve (model KSV8WA [XIAMEN KOGE MICRO TECH CO., LTD, Taiwan, R.O.C.]) in the switching section and the SSR (Solid State Relay), which switches the pump (model VP-6035S [Techno Takatsuki, JAPAN], its output pressure is 20 kPa, and its exhaust volume is 20 L/min) on and off. The microcontroller also controls the air supply and exhaust to the airbag to achieve the specified tilting angle by detecting the tilting angle with the angle sensor. When supplying air, the solenoid valve is switched so that the air on the output side of the pump passes through to the desired airbag, and then the pump is operated to supply air to the desired airbag, and the pump is stopped when the desired tilting angle is reached. To prevent airbag rupture due to excessive air supply, three pressure sensors (pressure sensors 1 − 3) are provided to monitor the pressure of each airbag, and if the detected maximum pressure (set at 20 kPa) of these pressure sensors is exceeded, the pump stops to ensure safety. When exhausting air, the solenoid valve is opened so that the air circuit on the suction side of the pump is connected to the desired airbag, and the pump is operated to suction air. The pump stops when pressure sensor 4 on the suction side of the pump reaches a negative pressure (set at − 10 kPa) as a confirmation that air has been completely exhausted from each airbag. The airbag capacity for tilting is approximately 7 L and the airbag capacity for support is approximately 2 L. An air pump actuator, with a sound volume of 40 dBA (at 1 m) during operation, controls the Z-shaped tilting unit, which has external dimensions of 380 mm in depth × 255 mm in width × 180 mm in height with a weight of approximately 7.4 kg. The air pump actuator is located near the foot of the bed and can be used with a home alternate current power supply of 100 V, 50/60 Hz. The rotating direction of the movable plate can be changed by inflating and contracting the air bag, whereby the movable mattress attached to the movable plates rotates. Therefore, the movable mattress can be gradually tilted to one or the other side and can also be flattened by inflating or deflating the small airbags (Fig. 5).

**Figure 6:**
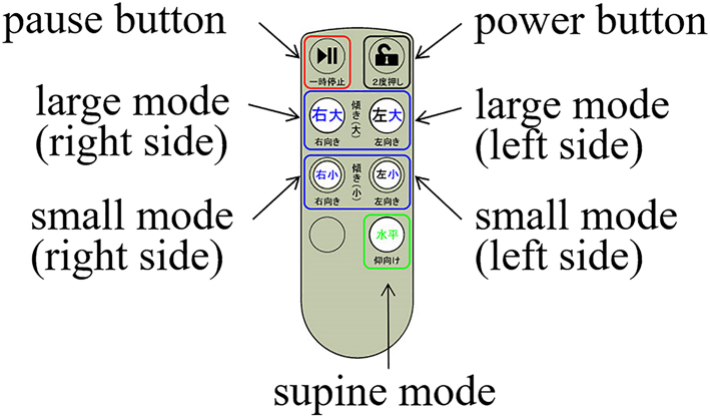
Remote control. Four buttons control tilting: large mode (right and left sides) and small mode (right and left sides). Press the supine mode button to change to the supine position, and press the pause button to stop tilting.

The tilt angle is controlled in the range of 0° to 60° by converting data from the angle sensor attached to the upper- centre part of the movable plate. The tilting angle was calculated from the direction of gravity based on the detected values of the MPU-6050 tri-axis acceleration sensor (TDK corporation, JAPAN) attached to the upper movable plate. If the sum of the measured acceleration values of the three axes is not 1 G, the sensor is judged to be faulty, and the power turned off. The error is assumed to be within ± 2%. The tilting angle can be programmed based on the type of nursing care or the patient’s characteristics (such as body weight or body conditions). Caregivers can easily operate the system by pressing once one of four buttons on the remote control. For example, the angle can be set to 45° during nursing care (diaper changing) and 20° during rest time (i.e., large (45°) left, large (45°) right, small (20°) left, and small (20°) right; Fig. 6). When the tilting button is pressed once, it tilts at a constant speed up to the pre-set angle. When the supine mode button is pressed once, air is released from the airbag and the movable plate moves into the horizontal position; thus, the patient’s body is in the supine position. The tilting stops when the pause button is pressed once. The remote control can be locked to prevent erroneous operation and released by pressing the power button twice.

The tilting speed is controlled by the airflow rate inside the airbag controls, which can also be programmed beforehand. The maximum slow speed is 17 s for a 45° tilt.

## Evaluation of the Programmable Lateral Position Changer

### Aim

This study aimed to objectively and subjectively evaluate the device from a caregiver’s perspective.

### Methods

#### Study design

**Figure 7:**
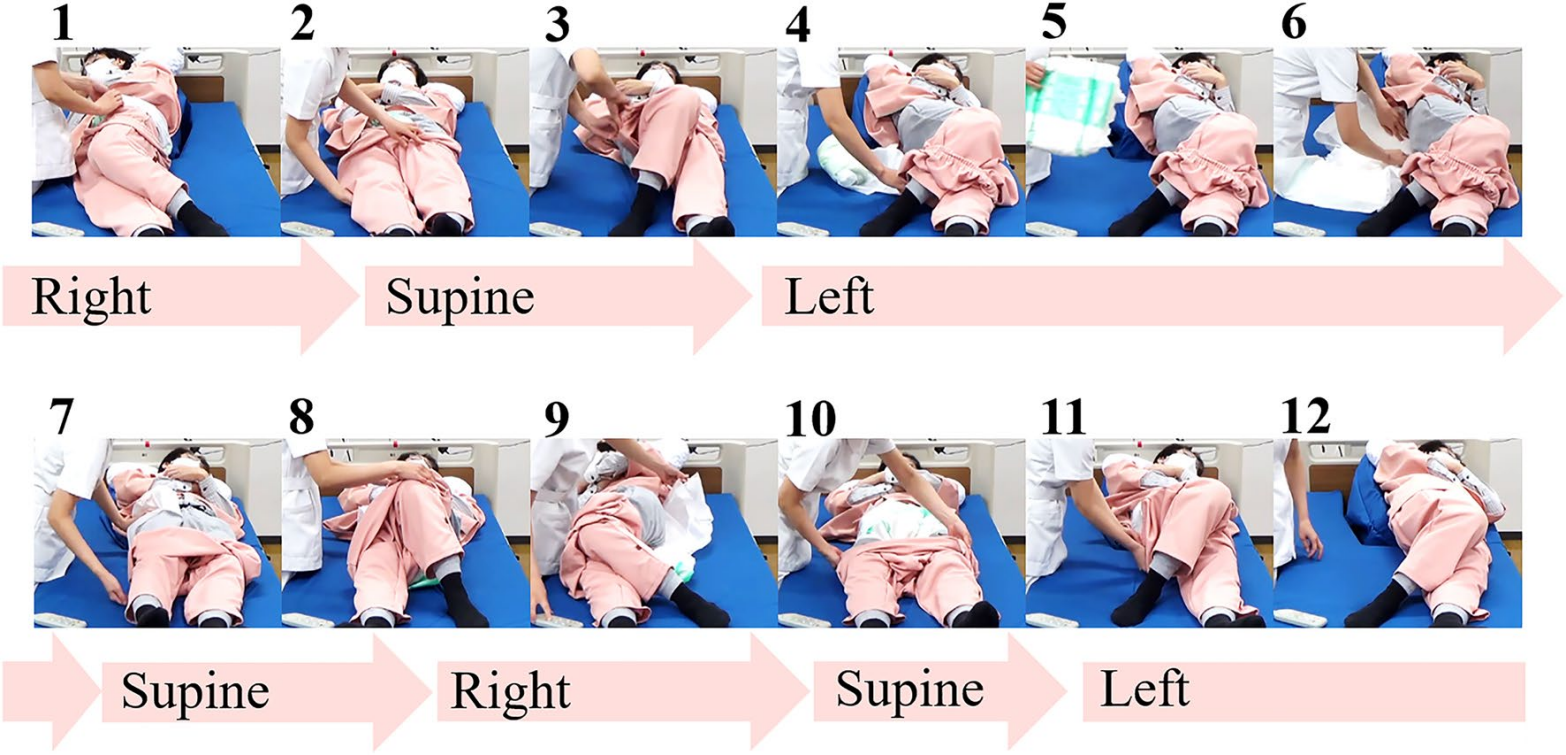
The simulated turning procedures. The nursing care procedure was performed follows: participants (caregivers) laterally turned a care receiver from the right lateral decubitus position (1) to the left lateral decubitus position (11, 12) to simulate the process of changing a patient’s diaper (2–10).

This was a comparative study with a randomised crossover design. We compared the outcomes between two care methods: care using the prototype by one caregiver (hereafter ‘Device method') and conventional care without the device by one caregiver (hereafter ‘Manual method’). The nursing care procedure was as follows: participants (caregivers) laterally turned a care receiver from the right lateral decubitus position to the left lateral decubitus position to simulate the process of changing a patient’s diaper (Fig. 7).

There were two sequences for performing the two care methods: one sequence is the Device method, which is first and the Manual method, which is second, and the other sequence is reverse. All participants performed both the Device and Manual methods once in either sequence. Participants were randomly assigned at a 1:1 ratio to a sequence according to their arrival order at the experiment room. For randomisation, we used a randomised allocation table that generated random numbers using a software program operated by an independent researcher. The table for random allocation was sealed and kept in a locked drawer until the day of the experiments. An independent researcher enrolled participants, assigned them to interventions, and explained the procedure for using the device. Therefore, blinding was not possible in this study. There was no important change in the methods after the study commencement, including eligibility criteria.

Additionally, we used a 3-min interval between the two interventions as the washout time to avoid carrying over the caregiver’s physical burden to the second intervention. According to previous studies^20–22^, the heart rate of caregivers indicates their physical burden, and the heart rates were 128 and 95 bpm while turning and changing pads, respectively^23^. In addition, the heart rate recovery time from peak heart rate to baseline heart rate, was a minimum of 3 min^24,25^. Based on these studies, we set a 3-min interval as a washout time.

#### Participants

We included care workers and nurses aged 20–60 years, with clinical experience in lateral turning and changing diapers who worked at the facility where the study was conducted in Osaka, Japan. We excluded care workers and nurses with health conditions that might have interfered with the care protocol and those with severe low back pain, which might have worsened by participating in the study.

#### Settings

The interventions were performed in a simulated patient room at the facility between November and December 2020. The standard bed frame was equipped with an electric switch that raised the bed height, and the left and right bed fences could be unlocked by being pulled out; the width of the mattress on the bed frame was 83 cm. The height of the bed (from the floor to the mattress surface) at the beginning of the intervention was 68 cm. Participants could freely change the height of the bed and remove the bed railing. At the end of the intervention, the bed and beddings were reset to default settings. The care receiver was a healthy man in his 60 s, weighing 65 kg, was 175 cm tall, and acted as a dependent patient. To ensure that he faithfully represented a bedridden patient with contractures, communication difficulties, and no limb movement, he was instructed to provide no assistance to caregivers and to relax his muscles. In addition, the researcher explained the purpose of the study and the mechanism and features of the device to the care receiver beforehand, and the care receiver was confirmed to behave as a bedridden patient by lying on the device. We used a simulated—instead of a real—patient to exclude possible biases caused by patient variation and because this was a feasibility study, which aimed to evaluate caregiver load; however, it was important that caregivers provide the care to a real person so that their power or touch of hands for turning the care receiver would be different from that for a mannequin. The device used in the study, along with a personal computer on which a video on how to use it could be viewed, was installed in a conference room in the facility where the research participants worked one week before the study was conducted so that anyone could freely practice the device. In addition, on the day of the study, we provided participants with instructions regarding the nursing care procedure and how to operate the device at the bedside.

#### Outcomes

##### The trunk flexion angle

*Previous studies*

One of the causes of low back pain is irreversible damage to the lumbar intervertebral discs due to excessive force on the discs^26^, and lumbar intervertebral disc pressure is considered to be a physical quantity that directly indicates the force on these discs. However, since intradiscal pressure measurement requires the use of an invasive procedure of inserting a pressure sensor directly into the disc^27^, a pressure estimation method based on a biomedical model is currently used^28^. The upper limit of 3400 N was defined in the Work Practices Guide for Manual Lifting^29^. The guideline was based on the autopsy data of previous studies^30,31^ and has since been reinforced by epidemiologic studies and other sources^32^. Today, the criterion of 3400 N is a widely recognised standard, although significant differences by age and sex have been reported^33^. This criterion has also been used in studies of patient handling in the healthcare field, and it has been shown that the low back load on caregivers during patient handling, such as repositioning and transfers, is extremely high^34,35^.

Forward bending is one of the most common causes of load on the lumbar intervertebral discs. According to Nachemson’s study^36^, in the standing position, forward flexion of 20***°*** causes disc loading. According to Andersson’s study^37,38^, as the flexion angle increases, lumbar intervertebral disc pressure also increases, and muscle activity stops at 45***°*** of trunk flexion angle. Based on the estimated spinal compression forces, unloaded trunk flexion at a 45***°*** angle causes compression forces to exceed 50 percent of the NIOSH threshold^39^. Furthermore, carrying a light load (100 N) significantly increases the magnitude of spinal compression during trunk flexion, exceeding 85 percent of the NIOSH threshold at a 45***°*** trunk angle^39^. Based on these basic biological studies, a trunk flexion angle of 45***°*** or more was defined as an extremely hazardous posture and a high risk of low back pain in the workplace^40,41^. Punnet^42^ conducted a case–control study based on this threshold and found an increased risk of low back pain in workers with a non-neutral posture of 45***°*** or more. Studies evaluating the risk of low back pain for caregivers have also been conducted using a threshold based on trunk flexion^43–45^.

*Trunk flexion angle in this study*

The primary outcome was the time it took for caregivers’ trunk flexion angle to exceed 45°. We defined the ‘trunk flexion angle’ as the angle between the vertical line and a straight line passing through the greater trochanter and shoulder. Trunk flexion angle was estimated using inertial measurement units (AHRS IMU sensor [WT901BLECL], WitMotion Shenzhen Co., Ltd). The sensor was a small (51.3 × 36 × 15 mm), wireless, battery-powered unit that measured and recorded acceleration (triaxial, ± 16 g) and angular velocity (triaxial, ± 2000°/s). A trunk postural data sensor was placed on each participant’s manubrium sterni to minimise the interference of the device during work activities. The device logged data at a rate of 5 samples/s. We also incorporated video analysis to enhance the quality of the trunk flexion angle data^46–48^. The correction angles were calculated using linear regression between sensor data and the trunk flexion angle on several pictures obtained from the videos using the Kinovea software (Kinovea Inc). For easy and precise measurement, we attached the tracking markers on the acromion and greater trochanter of participants. To establish inter-rater reliability, angle measurements using Kinovea were performed by two independent testers who were not involved in this study. The inter-rater reliability of these two testers was 97.5%. We derived a regression equation using the sensor and some video images during the first intervention for each participant. Subsequently, using the regression equation, we computed the corrected angles, and the coincidence of angles between the corrected angles and the angles from video images was confirmed during the second intervention when the slope of the regression curves was 1.0. We confirmed the slopes of all regression curves and the coefficients of determination of all regression equations, and a well-fitted model was constructed.

Based on previous studies^39,42,44,45^, we established a trunk flexion angle of 45° as the threshold level of low back load to evaluate the efficacy of our novel assistive device. We categorised and calculated the time of flexion (time and %time), the average and maximum trunk flexion angle during the turning intervention, the frequency of trunk flexion maintenance at > 45°, and the maximum trunk flexion maintenance time at > 45°.

##### Working time

Working time was defined as the time (in seconds) required to perform a complete turning intervention.

##### Subjective fatigue

Before and after each intervention, participants reported subjective fatigue (body fatigue and low back fatigue), measured using a 100-point visual analogue scale (VAS) with 0 points indicating ‘no fatigue’, and 100 points indicating ‘unbearable fatigue’. In the analysis, the change (range from − 100 to 100) calculated by subtracting the post-intervention value from the pre-intervention value is used. Thus, if the change is negative, it indicates a decrease in subjective fatigue after the intervention compared to before the intervention.

##### Comparative evaluation and usability

Upon the completion of both interventions, participants performed a comparative evaluation of the usability of the device via a survey. They evaluated the device’s usefulness in labour-saving *(care using the device allows less workload for caregivers compared to the manual method.)*, having both hands of the caregiver free for working *(care using the device allows easier care, as a result of both caregivers’ hands being free compared to the manual method.)*, and comfort for care receivers *(care using the device is more comfortable for care receivers compared to the manual method.),* in comparison with the manual method by rating according to a seven-point Likert scale (from strongly agree to strongly disagree). Furthermore, participants rated items, including the tilt angle *(what do you think about this device’s tilt angle for ease of patient care: proper, steep or shallow?)* and tilting speed*(what do you think about the tilting speed for this device: proper, too slow, or too fast?)*, with additional comments.

#### Statistical analysis

Demographic data were analysed using descriptive statistics and are reported as means with percentages or frequencies for categorical variables. The comments that accompanied the usability scoring survey were classified according to similarity. After classification, the relevance of the classification was checked by an independent researcher. To evaluate whether the device was effective in reducing caregiver burden, we analysed continuous variables using linear mixed models, with intervention for repeated measures for each participant. Intervention, period, and intervention-by-period were treated as fixed effects, and participants were treated as random effects. We estimated the adjusted differences of variables by classifying the following three patterns:

・There was a significant difference in intervention-by-period (regardless of the period); the adjusted difference was estimated using a model that included intervention, period, and intervention-by-period.

・There was no significant difference in intervention-by-period, but there was a significant difference in period; the adjusted difference was estimated using a model that included intervention and period.

・There was no significant difference in intervention-by-period or period; the adjusted difference was estimated using a model that included only the intervention.

Analyses were performed using JMP® 15 (SAS Institute Inc., Cary, NC, USA). The level of statistical significance was set as p < 0.05.

#### Sample size

Based on a pre-test involving three experiments, the mean times (standard deviation [SD]) in which the trunk flexion angle exceeded 45° was 31.5 (30.0) s and 53.4 (2.2) s for the Device and Manual methods, respectively. We set the correlation during the study periods to zero to preserve the power of the test. As this study did not include actual patients, the sample size calculations did not violate any ethical issue. Assuming a power and type 1 error in a two-sided test of > 0.8 and < 0.05, respectively; the minimum estimated sample size required for comparison between the Device and Manual methods was 32. Moreover, we considered the findings of previous studies that evaluated low back loads by measuring trunk flexion angle using sensors in healthcare workers^11,44,49^ and a comparative study on device development^50^. Based on the results of the pre-test and these previous studies, we set the target sample size to 30.

#### Registration number and name of study registry

The study was registered with the University Hospital Medical Information Network (registration number: UMIN000037746). All methods were carried out according to relevant guidelines and regulations.

#### Ethical considerations

All participants provided oral and written informed consent before the experiment, and the experimental protocol was approved by Osaka University Clinical Research Review Committee (approval number: TI 19,097).

### Results

#### Participants’ Information

We allocated 30 participants to the interventions in this study; 15 participants were allocated to each of the two sequences. We included 28 participants in the final analysis after excluding two participants who did not report their demographic characteristics; in addition, one of these participants did not have the sensor fastened, causing an error in data collection, i.e., this participant met two exclusion criteria. Adverse events were not observed during this study. The summary of participants’ data is shown in Table 1.

**Table 1:** Characteristics of participants.

Variable	Category	Value
Sex, n (%)	Female	19 (67.9)
	Male	9 (32.1)
Occupation, n (%)	Care workers	24 (85.7)
	Nurses	4 (14.3)
Age (years)		41.2 (11.5)
Healthcare worker experience (years)		9.1 (5.8)
Height (cm)		161.6 (8.8)
Frequency of assistive device use, n (%)	Less than once per month	24(85.7)
	Approximately 10 times per month	1(3.6)
	More than once per week	0(0.0)
	Almost every time	2(7.1)
	No answer	1(3.6)
Frequency for care by multiple caregivers, n (%)	< 1%	7(25.0)
	1–9%	9(32.1)
	10–29%	4(14.3)
	30–49%	1(3.6)
	> 50%	2(7.1)
	No answer	5(17.9)

Age, healthcare worker experience, and height are presented as mean (standard deviation).

#### Outcomes and estimation

All variables were analysed using linear mixed models with intervention for repeated measures for each participant, and least-square means (LSM) and 95% confidence intervals (CIs) are shown in Table 2. The crude mean of outcomes for the Device and Manual methods are shown in Supplementary Table S1, and sensibility analysis via a linear mixed model is shown in Supplementary Table S2.

**Table 2:** LSM and differences between the device and manual methods using a linear mixed model.

Dependent variables		D		M		diff	SE	95% CI		*t*-value	*p*-value
		LSM	SE	LSM	SE			Lower	Upper		
Time of trunk flexion [s]	(> 45°)	77.1	9.8	103.5	9.8	26.5	6.1	14.1	38.9	4.37	< .001
Time of trunk flexion [%]	(> 45°)	29.0	4.1	52.0	4.1	23.0	3.2	16.4	29.6	7.15	< .001
Average trunk flexion angle [°]		34.5	1.2	44.1	1.2	9.6	0.8	7.9	11.3	11.78	< .001
Maximum trunk flexion angle [°]		65.9	2.3	72.1	2.3	6.2	1.8	2.5	10.0	3.45	0.002
Frequency of trunk flexion maintenance	(> 45°, > 4 s)	5.8	0.8	7.1	0.8	1.4	0.6	0.0	2.7	2.10	0.045
Maximum flexion maintenance time [s]	(> 45°)	11.3	2.6	20.6	2.6	9.4	2.7	3.7	15.0	3.41	0.002
Working time [s]		263.6	9.6	198.0	9.6	− 65.6	9.1	− 84.1	− 47.0	− 7.24	< .001
Body fatigue		− 6.6	3.2	17.2	3.2	23.8	4.0	15.5	32.1	5.88	< .001
Low back fatigue		6.9	5.0	17.3	5.0	10.4	7.1	− 3.8	24.5	1.47	0.148

*D*, Device method; *diff*, differences; *LSM*, least squares mean; *M*, Manual method; *SE*, standard error.

##### The trunk flexion angle

The adjusted LSM time for extreme trunk flexion (> 45°) was 77.1 s for the Device method and 103.5 s for the Manual method. Thus, the time for extreme flexion was 26.5 s shorter using the Device method than using the Manual method (95% CI 14.1 s, 38.9 s) (*p* < 0.001). The adjusted differences in other parameters for trunk flexion were also significant, as was the time for extreme flexion.

##### Working time

The adjusted LSM working time was 263.6 s for the Device method and 198.0 s for the Manual method. Thus, the mean working time in the Device method was 65.6 s longer than that in the Manual method (95% CI: − 84.2 s, − 47.0 s) (*p* < 0.001).

##### Subjective fatigue

The adjusted LSM body fatigue score was -6.6 for the Device method and 17.2 for the Manual method. Thus, the body fatigue score in the Device method was 23.8/100 points lower than that in the Manual method (95% CI: 15.5, 32.1) (*p* < 0.001).

The adjusted LSM low back fatigue score was 6.9 for the Device method and 17.3 for the Manual method. Thus, there was no significant difference between the Device and Manual methods in terms of the VAS score for subjective low back fatigue (95% CI: − 3.8, 24.5) (*p* = 0.148).

##### Comparative evaluation and usability

**Figure 8:**
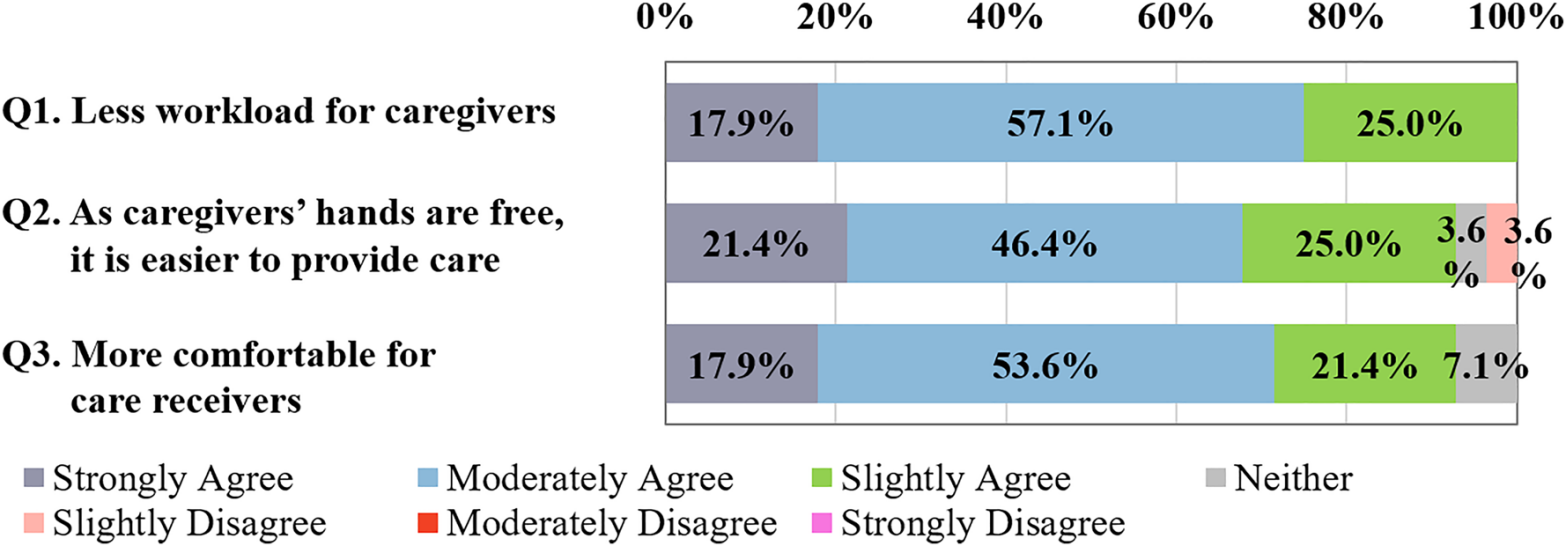
Comparative evaluation with the manual method (n = 28). Q1: Care using the device allows less workload for caregivers compared to the manual method. Q2: Care using the device allows easier care, as a result of both caregivers’ hands being free compared to the manual method. Q3: Care using the device is more comfortable for care receivers compared to the manual method.

As shown Fig. 8, compared to the manual method, most participants positively evaluated the device: all (Strongly Agree: 17.9%, Moderately Agree: 57.1%, and Slightly Agree: 25.0%) participants reported that it could reduce workload, and 92.8% (Strongly Agree: 21.4%, Moderately Agree: 46.4%, and Slightly Agree: 25.0%) felt care was easier because they could use both hands during care provision, which improves the comfort of care receivers.

Of all participants, 46.4% felt the angle was appropriate for patient care, whereas 35.7% felt the angle was too steep and risky, and 17.9% felt the angle was too shallow, making it difficult to place a diaper under the patient’s body (Table 3). Regarding tilting speed, 53.6% of the respondents felt the speed was appropriate, whereas 46.4% felt it was too slow for care and too prolonged for turning and repositioning.

**Table 3:** Usability of the device (n = 28).

What do you think about this device’s tilt angle for ease of patient care? (n = 28)			
	n	%	Reason (free response)
Proper	13	46.4	Care is easy (6) Too close to the side rail (5) Risk of falling out of bed (3) Difficult to insert diaper under the patient's body (2) Concern for the patient with contracture (1)
Steep	10	35.7	
Shallow	5	17.9	
What do you think about the tilting speed for this device? (n = 28)			
	n	%	Reason (free response)
Proper	15	53.6	Too slow for care (5) Too slow for me but proper for care receiver (4) Appropriate or proper because a high speed may worsen or affect patients’ conditions (4) It takes too much time from left lateral decubitus to right via supine position (2) Appropriate or proper because a high speed may pose a risk the patients (1) Want to customise for patient conditions (1)
Too slow	13	46.4	
Too fast	0	0	

### Discussion

We developed a prototype of novel technology, the Programmable Lateral Position Changer, and performed both objective and subjective evaluations from the perspective of the caregivers.

To measure our primary outcome of trunk flexion angle among caregivers during patient care, we analysed the trunk flexion angle categories using 45° as the threshold, based on previous studies ^39,42,44,45^. In this study, the time of trunk flexion exceeding 45° improved by 26.5 s (23.0%) with the use of the device compared with that in the manual group. The average and maximum trunk flexion angles of caregivers during patient care with the device were smaller than those observed with the manual method; moreover, the frequency of trunk flexion maintenance and the maximum flexion maintenance time was reduced with the device. Hence, the device was found to potentially reduce the low back load of caregivers. However, even with the use of the device, the trunk flexion angle of the caregiver is sometimes 45° or more. In addition, even if there are times when the flexion is less than 45°, the caregiver still suffers from a lumbar load, even with a slight forward tilt of 20°, as evidenced by Nachemson's study^27^. In other words, the new device has not completely eliminated the caregiver’s low back load. Indeed, it is clear that the current device reduces the low back load by decreasing the severe trunk flexion time; however, it is not clear at this time whether this prevents the development of low back pain. According to the review examining the effectiveness of assistive devices in preventing low back pain, there is no clear evidence to support the effectiveness of assistive devices in preventing low back pain^51^. This is partly because the magnitude of the load on the lumbar region is not only factor related to the onset of low back pain, and partly because the relationship of low back pain with the proper use of the device has not been clarified. In order to verify the effectiveness of this device in preventing low back pain in the future, it is necessary to conduct a long-term study on the use of the device after its introduction, and the resulting prevalence of low back pain.

Second, changes in the subjective body fatigue level of the caregivers exceeded 20 points between the device and manual interventions. Regarding the VAS scores, Jaeschke et al.^52^ described the importance of the minimal clinically important difference (MCID) as “the smallest difference in scores in the domain of interest which patients perceive as beneficial”. In the results of VAS measurements of changes in chronic low back pain^53^ and acute pain^54^, the MCID ranged from 13 to 19 mm. Although the participant demographics were different from those in this study, we considered the difference of 26.4 in current study to be clinically significant. Therefore, the developed device effectively assisted caregivers during patient care by reducing subjective body fatigue levels. In addition, the change in subjective fatigue with the device method was negative, indicating less fatigue after the intervention than before. This trend was the same in periods 1 and 2 of the intervention (see Table S1). Although it is generally unlikely that the caregivers' fatigue was reduced despite the care they provided, this study evaluated fatigue subjectively. Therefore, caregivers may have felt as if they were taking a break when using the device compared to their busy daily work since they only need to observe the care receiver while the device is slowly tilting the care receiver’s body. However, the difference in subjective low back fatigue level was not statistically significant. This result might have either been owing to the short washout period or small sample size for this variable.

Third, the working time in the current study was prolonged in the group using the device; similarly, previous studies have also found that the use of assistive devices prolonged the working time^55,56^. Prolonged working time may be a major reason for caregivers’ reluctance to use assistive devices. Even if these devices are effective in reducing caregivers’ low back loads and fatigue, the prolonged working time may reduce the frequency of use of this potentially novel device. It is necessary to shorten the total working time required to use the device. Insufficient experience with the device may be a major reason for this prolonged time, which may be reduced by effective training with the device.

Fourth, in terms of the labour-saving and the care receiver’s comfort, compared to the manual method, most participants positively evaluated the device, and indicated that the device would be beneficial to many caregivers. Caregivers’ assessment of the tilt angle of the moving mattress varied. While nearly half of the participants found it appropriate, there were some concerns regarding the care receivers’ safety, especially the patient’s body being too close to the bed’s side railing when it tilted. The Programmable Lateral Position Changer tilts the patient’s body slowly, and the body hardly slides laterally during tilting. Therefore, when most participants used the device, the patient’s body did not move horizontally to the side in advance. However, in manual turning, the patient’s body slides more laterally; hence, most caregivers move the patient’s body horizontally toward them at the beginning of care to ensure the care receivers’ safety, preventing them from rolling into the side railing. Due to the difference in these procedures, the distance to the bed railing became closer than usual, and the caregiver perceived some risks; however, the caregivers do not bear the burden of moving the patient horizontally. Regarding the tilting speed, some participants stated that time constraints or shortcomings were acceptable, considering the safety and comfort of the care receivers. The gradual tilting of the device may contribute to minimising pain associated with forced postural change related to tilting. Quick manual tilting often causes considerable pain to care receivers with contracture; however, gradual tilting is almost impossible manually. It is important to utilise the device’s novel value for both caregivers and care receivers. The gradual tilting of the device also may prolong the working time. Efficient working procedures should be designed to utilise the novel device such that caregivers prepare clothes while the device gradually tilts.

As the ageing populations in many countries continue to grow^1,2^, there is an additional strain (including physical strain) on the nursing staff who care for older patients with special needs. Developing an effective device for use in hospitals and nursing homes for turning and supporting bedridden patients will improve and maintain care quality. It is necessary to continue seeking multi-faceted solutions for reducing caregiver load and continuously develop this new system, despite the many challenges in the practical application of this device, such as safety concerns, high development cost, and usability.

### Strengths and limitations

This study’s findings are meaningful, as it was conducted using an appropriate study design with randomised interventions. Furthermore, analyses were performed using a mixed model that enabled comparisons within each participant, thereby avoiding the influence of participant characteristics, such as the caregivers’ clinical experiences or sex. However, the study has some limitations. First, care receivers could not be evaluated, because only one care receiver was involved in the study to ensure a uniform level of task burden for all participants. Although caregivers are primary users, care receivers are also essential users of such assistive devices; hence, their opinions regarding comfort and safety must also be recognised. Furthermore, in this study, because a healthy person played the role of a bedridden patient, the condition of actual paralysis and contractures may not have been faithfully represented. Therefore, it cannot be denied that the caregiver’s evaluation of the device may change when using this bed to care for patients with contractures. Similar to our study, other studies have simulated healthy people when measuring the effectiveness of turning and lateral transfer devices in an experimental setting^15,17,57^. However, when our product is completed, it should be used on patients with contractures in a clinical setting to verify the safety of the devices. Second, it used a small sample size and convenience sampling. The number of valid answers to the questionnaire was small (28), and participants were recruited from only one facility. Therefore, detailed analyses, such as a stratified analysis based on age and years of experience, which may affect the usability and usefulness, were not performed. Therefore, our participants may not be representative of the broader nursing population, which limits the generalisability of our results. Finally, the device used in the study was set up in a conference room at the study facility in advance and was available for practice at will, but when the participants were asked on the day of the study, few had practised with the device and most had only watched a video of it. Lack of proficiency in using the device may have been related to longer working time and increased subjective fatigue. In the future, it will be necessary to assess the degree of proficiency of the participants and consider the effects on the evaluation.

### Conclusion

We developed a novel device that was programmed to laterally turn (on the long axis of the body) and support a patient’s body while receiving care. The new assistive device reduced the low back load and subjective fatigue level in caregivers, and received a positive subjective evaluation.

## Supplementary Information

Supplementary Information.

## Data Availability

The dataset used and/or analysed during the current study are available from the corresponding author on request.
