# Supplementary material for: Development and evaluation of a new assistive device for low back load reduction in caregivers: an experimental study
Source: Sci Rep. 2022 Nov 9;12:19134. doi: 10.1038/s41598-022-21800-5 (PMC9646712; doi:10.1038/s41598-022-21800-5)
Supplement: Supplementary file 1 — Supplementary Information. [file 41598_2022_21800_MOESM1_ESM.pdf]

Development and evaluation of a new assistive device for low back load reduction in caregivers: an experimental study

Yuka OMURA [1,2], Masayuki HIRATA [3,4], Toshiki YOSHIMINE [4,5], Eiji NAKATANI [6,7], Tomoko INOUE [1]

[1]Division of Health Sciences, Osaka University Graduate School of Medicine, Osaka, Japan.

[2]Global Center for Medical Engineering and Informatics, Osaka University, Osaka, Japan.

[3]Department of Neurological Diagnosis and Restoration, Osaka University Graduate School of Medicine, Osaka, Japan.

[4]Endowed Research Department of Clinical Neuroengineering, Global Center for Medical Engineering and Informatics, Osaka University, Osaka, Japan.

[5]Iseikai Medical Corporation, Osaka, Japan.

[6]Graduate School of Public Health (Medical Statistics), Shizuoka Graduate University of Public Health.

[7]Division of Clinical Biostatistics, Research Support Center, Shizuoka General Hospital, Shizuoka, Japan.

Supplementary table S1: Crude mean and standard deviation in each period and intervention

Dependent variables			D			M		
		Period	n	Mean	SD	n	Mean	SD
Time of trunk flexion [s]	(> 45°)	1	14	66.5	38.0	14	114.9	64.6
		2	14	87.7	46.1	14	92.2	53.2
		overall	28	77.1	42.8	28	103.5	59.2
% time of trunk flexion [%]	(> 45°)	1	14	25.3	13.6	14	54.4	26.0
		2	14	32.8	17.1	14	49.6	27.0
		overall	28	29.0	15.6	28	52.0	26.1
Average trunk flexion angle [°]		1	14	33.8	5.6	14	44.5	7.8
		2	14	35.2	6.2	14	43.7	6.9
		overall	28	34.5	5.8	28	44.1	7.2
Maximum trunk flexion angle [°]		1	14	65.0	12.5	14	73.1	11.3
		2	14	70.8	9.0	14	75.2	12.8
		overall	28	67.9	11.1	28	74.1	11.9
Frequency of trunk flexion maintenance	(> 45°, > 4s)	1	14	5.1	3.2	14	6.5	3.9
		2	14	7.6	5.1	14	6.6	4.5
		overall	28	5.8	3.6	28	7.1	4.8
Maximum flexion maintenance time [s]	(> 45°)	1	14	9.6	5.6	14	26.5	22.2
		2	14	13.0	8.1	14	14.8	11.7
		overall	28	11.3	7.0	28	20.6	18.4
Working time [s]		1	14	255.0	32.1	14	218.3	68.4
		2	14	272.2	54.4	14	177.8	31.4
		overall	28	263.6	44.7	28	198.0	56.2
Body fatigue		1	14	-2.8	16.4	14	15.8	18.1
		2	14	-10.7	15.8	14	18.6	16.6
		overall	28	-6.6	16.3	28	17.2	17.1
Low back fatigue		1	14	6.9	13.1	14	17.3	24.8
		2	13	-14.3	18.2	14	18.1	16.6
		overall	27	-3.3	18.9	28	17.7	20.7

D, device method; M, manual method; SD, standard deviation.

Supplementary table S2: Sensibility analysis via a linear mixed model

Dependent variables		Independent variables	df	F-value	p-value
Time of trunk flexion [s]	(> 45°)	Period	1	0.01	0.907
		Intervention	1	6.21	0.018
		Intervention-by-period	1	1.42	0.245
% Time of trunk flexion [%]	(> 45°)	Period	1	0.17	0.683
		Intervention	1	12.60	0.001
		Intervention-by-period	1	0.66	0.424
Average trunk flexion angle [°]		Period	1	0.12	0.734
		Intervention	1	18.14	<.001
		Intervention-by-period	1	0.23	0.635
Maximum trunk flexion angle [°]		Period	1	4.92	0.036
		Intervention	1	3.48	0.070
		Intervention-by-period	1	0.22	0.641
Frequency of trunk flexion maintenance	(> 45°, > 4s)	Period	1	0.11	0.747
		Intervention	1	2.57	0.117
		Intervention-by-period	1	0.69	0.414
Maximum flexion maintenance time [s]	(> 45°)	Period	1	2.36	0.137
		Intervention	1	10.99	0.002
		Intervention-by-period	1	3.01	0.094
Working time [s]		Period	1	1.70	0.203
		Intervention	1	3.90	0.055
		Intervention-by-period	1	3.15	0.088
Body fatigue		Period	1	0.38	0.544
		Intervention	1	8.58	0.005
		Iintervention-by-period	1	1.20	0.284
Low back fatigue		Period	1	3.80	0.062
		Intervention	1	2.15	0.148
		Intervention-by-period	1	5.09	0.033

D, device method; M, manual method; SD, standard deviation; df, degress of freedom.
